# Shear bond strength of orthodontic metal brackets to aged composite using three primers

**DOI:** 10.4317/jced.53731

**Published:** 2017-06-01

**Authors:** Ali Tayebi, Farnoosh Fallahzadeh, Marzieh Morsaghian

**Affiliations:** 1 Assistant Professor of Orthodontics, Dental Caries Prevention Research Center, Qazvin University of Medical Sciences, Qazvin, Iran; 2 Assistant Professor of Operative Dentistry, Dental Caries Prevention Research Center, Qazvin University of Medical Sciences, Qazvin, Iran; 3 Postgraduate Student of Orthodontics, Dental Caries Prevention Research Center, Qazvin University of Medical Sciences, Qazvin, Iran

## Abstract

**Background:**

This study aimed to assess the effect of surface preparation with sandblasting and diamond bur along with the use of three primers on shear bond strength (SBS) of metal brackets to aged composite.

**Material and Methods:**

In this *in vitro*, experimental study, 60 Filtek Z250 composite discs were fabricated (10×2mm), immersed in distilled water for 24 hours and subjected to 5000 thermal cycles. They were randomly divided into two groups (n=30) of sandblasting with aluminum oxide particles for 10 seconds and surface roughening with bur. Each group was randomly divided into three subgroups (n=10) for use of Transbond XT, Assure Plus and Composite Primer. Metal brackets were bonded and the samples were stored in distilled water for 24 hours followed by 2000 thermal cycles. The SBS of brackets was measured and the adhesive remnant index (ARI) score was calculated. The data were analyzed by one-way ANOVA, t-test and Chi square test.

**Results:**

The difference in the mean SBS was not significant among the six subgroups.

**Conclusions:**

All combinations of primers and surface preparation methods provided adequately high SBS between brackets and aged composite surfaces. Considering the ARI scores, surface roughening by bur is superior to sandblasting.

** Key words:**Shear strength, composite resins, orthodontic brackets, aged composite, surface preparation.

## Introduction

Adequate bond between the orthodontic brackets and tooth or restoration surfaces is a prerequisite for a successful orthodontic treatment. The increasing number of adults with extensive dental restorations seeking orthodontic treatment highlights the importance of bonding procedure in orthodontic treatment (1). Orthodontists must be able to obtain a strong bond between orthodontic brackets and enamel or restorative materials such as composite, amalgam and porcelain. However, achieving an adequate bond to restoration surfaces is sometimes challenging (2).

The demand for tooth-colored restorative materials has greatly increased (3). The use of amalgam has decreased and most patients demand composite restorations due to superior esthetics (3-5). Composite resins are extensively applied for restoration of carious teeth, pit and fissure caries, abfraction defects, diastema closure, build up of peg laterals, restoration of incisal fractures and composite veneers (6). Thus, composite restorations are frequently found in buccal surfaces of maxillary incisors as well as posterior teeth.

Methacrylate groups play a major role in bonding of composite resin to a composite surface (7). They are found in the oxygen-inhibited layer of non-polymerized resin on the composite surface and allow incremental repair of composite. The bond strength between the new and old composite is equal to the cohesive strength of composite (8). However, aged, polished or saliva-contaminated composites do not have the afore-mentioned superficial methacrylate layer (9,10). The half-life of methacrylate groups at 37°C is only 50 hours (11). Therefore, surface characteristics of an aged composite surface are significantly different from those of a freshly applied composite (3).

Several surface preparation methods have been proposed to overcome the problems encountered for bonding of orthodontic metal brackets to aged composite (4), which are classified into two groups of mechanical and chemical surface preparation techniques. Mechanical methods include roughening of the composite surface with diamond bur or sandblasting. The chemical techniques include acid etching with phosphoric acid or hydrofluoric acid and application of different bonding resins (4-6,12). Use of bonding agents can significantly increase the bond strength of orthodontic brackets to composite restorations (13). On the other hand, in contrast to restorative dentistry, there is no need for a permanent bond in orthodontic treatment and bond strength in the range of 6-10 MPa would suffice for orthodontic purposes (14).

An acceptable bracket bonding system in orthodontic treatment must be able to resist forces applied by orthodontic wires as well as loads in the oral environment. Shear loads are among the most common and most destructive forces applied in the oral environment, which can cause debonding of brackets (15). Some researchers have attempted to improve the bond strength of orthodontic attachments to amalgam and porcelain (16) but studies on methods to improve the bond strength of orthodontic brackets to aged composite restorations are scarce.

This study aimed to assess the SBS of orthodontic brackets to aged composite by use of three primers. The null hypothesis was that the SBS of orthodontic brackets to aged composite would not be significantly different by use of the three primers and different surface preparation methods.

## Material and Methods

This *in vitro* experimental study was conducted on 60 composite discs, which were randomly divided into two groups of sandblasting and diamond bur. Each group was randomly divided into three subgroups of 10 for use of Transbond XT, Assure Plus and Composite Primer. Sample size was calculated to be 10 in each subgroup considering α=0.05, β=0.2 and 80% study power.

Composite discs measuring 10mm in diameter and 2mm in thickness were fabricated of A3 shade of Filtek Z250 composite (3M ESPE, St. Paul, MN, USA). This composite is a methacrylate-based micro-hybrid composite containing zirconia-silicate particles measuring 0.01 to 3.5μm in size. All discs were fabricated by the same operator and light cured using Ortholux LED light curing unit (3M Unitek, Monrovia, CA, USA) with a light intensity of 950 mW/cm2, calibrated by a radiometer. Light curing was done from both sides for 40 seconds. The samples were then visually inspected to ensure absence of cracks or defects. The discs were stored in distilled water, incubated at 37±1°C for 24 hours (Dorsa, Tehran, Iran) and were then subjected to 5000 thermal cycles between 5-55°C with a dwell time of 30 seconds and transfer time of 4 seconds (Dorsa, Tehran, Iran) (17). Thirty composite discs were randomly selected and subjected to sandblasting by 50μ aluminum oxide particles (Korox Corundum, Bego, USA) from 10mm distance at 3.5 to 4.5 bar pressure for 10 seconds using a micro-etcher (Danville, CA, USA). The remaining 30 discs were roughened by a 008 fissure diamond bur (Brasseler, Savannah, GA, USA). Diamond bur was used with one back and forth motion in occlusogingival direction and one back and forth motion in mesiodistal direction. For every five discs, a new diamond bur was used (18). The surface of the discs was rinsed under running water and air-dried with oil-free air spray.

The discs in the sandblasting and diamond bur groups were randomly divided into three subgroups of 10 for the application of Assure Plus (Reliance Orthodontic Products, Itasca, IL, USA), Composite Primer (GC Dental Products, EUROPE) and Transbond XT (3M Unitek, Monrovia, CA, USA). Edgewise maxillary central incisor brackets (GAC, International, Bohemia, NY, USA) with 0.022-inch slot and 11.26 mm2 base area were used. The respective primer was applied on each disc according to the manufacturer’s instructions. Transbond XT (3M ESPE, St. Paul, MN, USA) composite was applied on the back of bracket and the bracket was then compressed on the disc surface. Excess composite was removed using the sharp tip of an explorer, and polymerization was performed using Ortholux LED light curing unit for 40 seconds (10 seconds from each of the mesial, distal, incisal and gingival sides). Characteristics of the primers and their method of application are shown in  Table 1 (18,19).

**Table 1:**
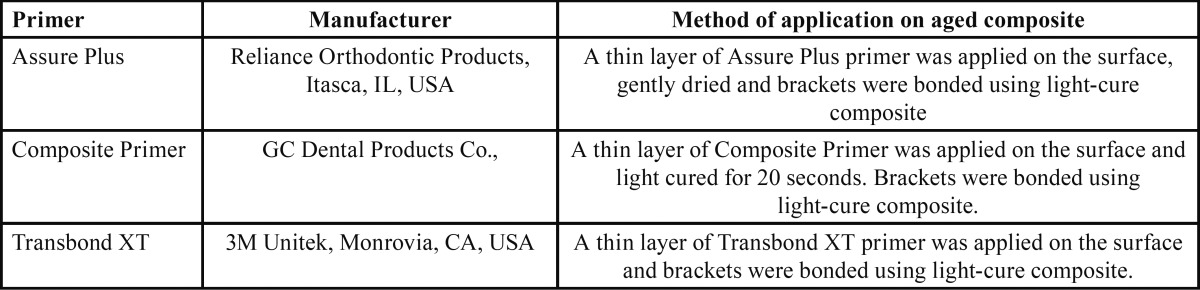
The primers used and their method of application as recommended by the manufacturer.

All samples were stored in distilled water at 37°C for 24 hours (Dorsa, Tehran, Iran) and were then thermocycled for 2000 cycles between 5-55°C with a dwell time of 20 seconds and transfer time of 4 seconds (5).

**Figure 1:**
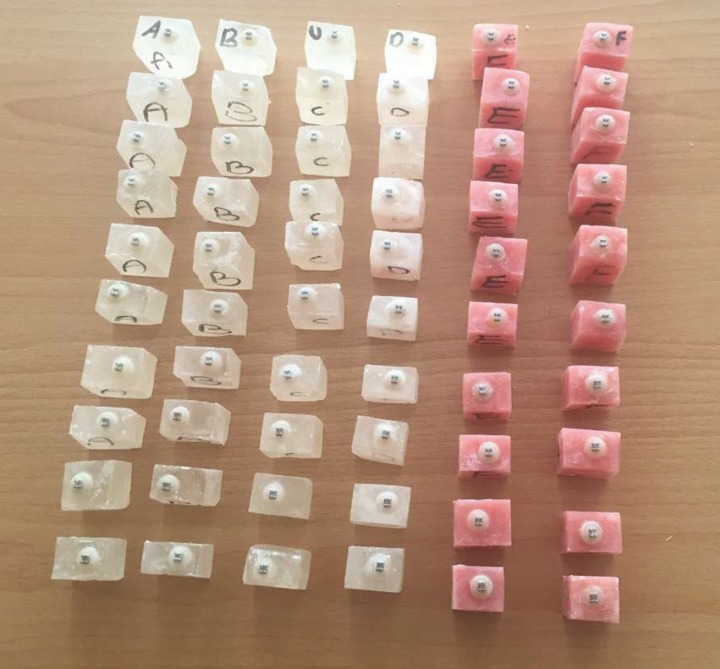
Mounted samples for bond strength testing.

For bond strength testing, wax boxes were fabricated and brackets were placed on top of them parallel to the longitudinal margins of the boxes. Auto-polymerizing acrylic resin was poured into boxes and the samples were embedded in acrylic resin in the boxes up to their upper margin. By doing so, the contact of acrylic resin and brackets was prevented and a proper stub was fabricated for transfer of samples to the universal testing machine (Fig. 1).

The SBS testing was performed in a universal testing machine (Zwick Roell GmbH & Co., Ulm, Germany). The samples were placed in the clasp in such a way that the bracket base was parallel to the vertical blade of the machine. The knife-edge blade mounted on the crosshead applied shear load to the wide part of bracket base at the adhesive interface in occlusogingival direction at a crosshead speed of 1mm/minute until failure. Maximum load at fracture was recorded in Newton. The value was divided by the bracket base (in mm2) to obtain the SBS value in MPa. Next, debonded samples were evaluated under a stereomicroscope (Carl/Zeiss Germany) at ×10 magnification for assessment of the mode of failure. The ARI score was also calculated using a four-point scale as follows (18):

Score zero: No adhesive remained on the restoration surface 

Score 1: Less than 50% of adhesive remained on the restoration surface 

Score 2: More than 50% of adhesive remained on the restoration surface 

Score 3: All the adhesive remained on the restoration surface 

The data were analyzed using SPSS version 20. The mean and standard deviation of SBS of metal brackets to composite surfaces were reported in the two groups and the six subgroups. The SBS of orthodontic brackets to aged composite surfaces was analyzed in the two groups and six subgroups using ANOVA. The t-test was applied to compare the SBS of primers in the diamond bur and sandblasting groups. Also, ARI scores were compared among the groups using Chi-square test. *P*<0.05 was considered statistically significant.

## Results

The results showed that the mean SBS of metal brackets to aged composite was 10.8±3.4MPa in the Assure Plus/bur, 13.8±5.2MPa in the Assure Plus/sandblasting, 7.57±4MPa in the Transbond XT/bur, 9.94±2.5MPa in the Transbond XT/sandblasting, 10.14±4.6MPa in the Composite Primer/bur and 10.95±6.7MPa in the Composite Primer/sandblasting sub-groups. According to one-way ANOVA, no significant differences were noted in SBS of the six subgroups (*P*=0.11).

Comparison of SBS of the three subgroups prepared with diamond bur ( Table 2) and the three sandblasted subgroups ( Table 3) revealed no significant differences either. Pairwise comparison of SBS with t-test revealed no significant difference between As-sure Plus/bur and Assure Plus/sandblasting subgroups (*P*=0.14), Transbond XT/bur and Transbond XT/sandblasting subgroups (*P*=0.13) or Composite Primer/bur and Composite Primer/sandblasting subgroups (*P*=0.75).

**Table 2:**
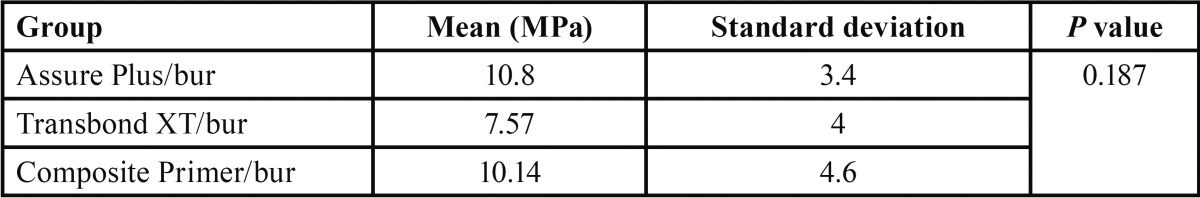
Comparison of shear bond strength of subgroups prepared with bur.

**Table 3:**
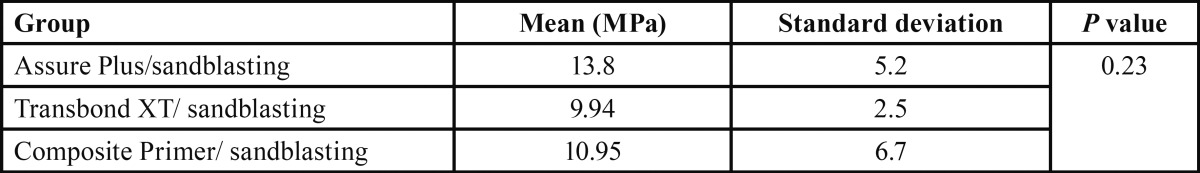
Comparison of shear bond strength of sandblasted subgroups.

The ARI scores of the six subgroups are shown in  Table 4. The ARI scores 0 and 1 were not seen in any subgroup. As seen in  Table 4, the highest ARI score seen in bur preparation group was score 3 (100% of adhesive remaining on the bracket base) while in sandblasted group, most samples showed fracture in composite base.

**Table 4:**
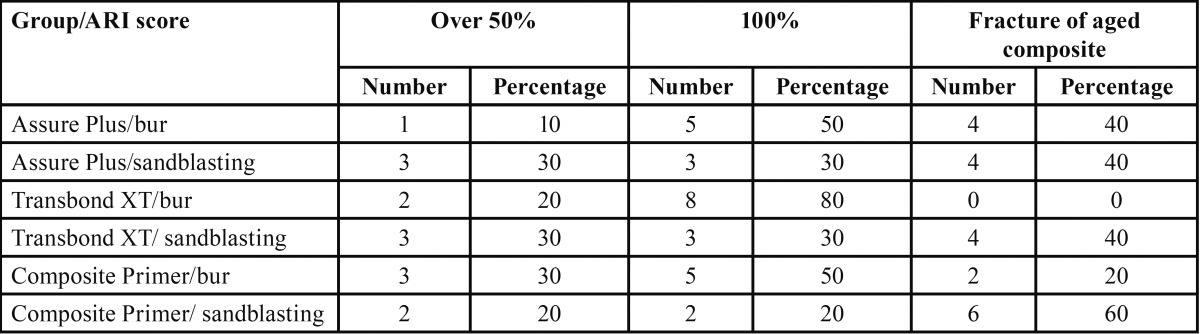
The Adhesive remnant index (ARI) scores in the six subgroups.

## Discussion

Bond of orthodontic attachments to composite restorations in the oral environment is similar to aging of the restoration in a humid environment for a long period of time. In this process, composite resin is saturated with water and its free radicals are no longer active. Absorbed water softens the matrix and results in formation of small cracks, resin resorption and debonding at the filler-matrix interface (20). Some studies have reported decreased bond strength between the old and new composites following the process of aging and storage in saliva (8).

Dental materials are subjected to mechanical, chemical and thermal stresses in the oral environment. Laboratory tests such as water storage and thermocycling are performed to simulate the clinical setting and assess the behavior of materials under these circumstances. Thermocycling is performed for artificial aging in order to assess the SBS of orthodontic metal brackets after aging (21). In the current study, aging was performed by water storage for 24 hours and thermocycling between 5-55°C for 5000 cycles at first and 2000 cycles later. Thermocycling accelerates the process of aging and water diffusion (22). Temperature difference between water baths in this process results in water sorption at the interface of the two materials with different coefficients of thermal expansion and eventually degrades the resin structure (23). Wide ranges of durations and temperatures of thermal cycles have been used in previous studies; however, all previous studies unanimously reported that thermocycling negatively affected the SBS (21,24). Some previous studies on the SBS of orthodontic brackets to composite did not perform aging for composite samples prior to bracket bonding (25); the bond strength values reported in such studies are often higher, which can be due to the use of fresh composite samples.

Several methods have been proposed to enhance the composite-composite bond. Surface roughening is one suggested technique to enhance the bond of a new composite resin to the matrix or filler particles of an old composite restoration (5). In the current study, the SBS of metal brackets to aged composite was assessed following sandblasting and surface roughening by bur along with the application of three primers. Based on the results, no significant difference was noted in SBS of the six subgroups. The order of SBS from the highest to the lowest was as follows: Assure Plus/sandblasting, Composite Primer/sandblasting, Assure Plus/bur, Composite Primer/bur, Transbond XT/sandblasting and Transbond XT/bur, respectively. The highest SBS values were noted in sandblasted samples in all six primer subgroups; however, the differences were not significant. Assessment of the results of ARI scores showed that most sandblasted samples experienced fracture in the composite base. Demirtas *et al.*, in 2015 reported the highest SBS following sandblasting with Al2O3 particles; they suggested this method to increase the bond strength of brackets due to minimal area of the prepared surface (22). Bayram *et al.*, in 2011 reported the highest bond strength following surface roughening by diamond bur. They used scanning electron microscopy and showed that sandblasting created areas of micromechanical interlocking while diamond bur created areas of both macro- and micromechanical interlocking and thus, the latter yielded greater retention than other methods (5).

The current study did not find any significant difference in SBS between sandblasting and bur preparation groups, and ARI scores showed higher frequency of fractures in the composite base in sandblasted group; thus, we recommended surface roughening by bur in the clinical setting since ARI scores revealed that samples prepared with bur mostly had ARI score 3 (100% of adhesive remained on the composite base); this type of fracture is optimal for orthodontic treatment. However, it should be noted that surface roughening by bur removes resin and exposes filler particles; thus, it may compromise the esthetics of the restoration, which is not favorable in anterior teeth (22). On the other hand, in order to prevent fracture or cracking of the surface, resin remnants should preferably remain on dental or restoration surfaces after bracket debonding (26). But, resin removal from dental surfaces following debonding is difficult and time consuming and may also damage the enamel or restoration surface.

In the current study, bond strength values in the two groups of sandblasting and bur preparation and their three subgroups of Transbond XT, Assure Plus and Composite Primer were sufficiently high and no significant difference was noted among them in this respect. This finding revealed that different compositions of the three primers did not significantly affect the SBS of brackets to aged composite. The MDP monomer is present in the composition of Assure Plus, which enables a chemical bond to enamel and dentin. This is one major advantage of this bonding agent to others. Also, presence of ethanol in this primer is another advantage, which enhances the bond to dentin. Since in the current study the adherent was hydrophobic composite base, presence of MDP and ethanol was not considered an advantage of Assure Plus compared to Composite Primer and Transbond XT and as seen in the results, no significant difference existed in SBS among the subgroups. Thus, it may be concluded that all three primers can be successfully used for bonding of metal brackets to aged composite. Considering the high cost of Assure Plus and Composite Primer, they are not recommended for bonding of metal brackets to aged composite restorations, and Transbond XT seems to be more cost-effective for this purpose.

At present, many orthodontic brackets are fabricated of stainless steel. These brackets have an easy fabrication process and are highly resistant to masticatory forces due to their optimal flexibility. Also, they are easily peeled off from the tooth surface and are affordable. However, composite resins cannot chemically bond to stainless steel; thus, retentive mechanisms such as bonding systems are required for this purpose (18). Due to the popularity of metal brackets and their common use in the clinical setting, this study was conducted on metal brackets.

In the oral environment, bonded brackets are subjected to shear, tensile, torsional or a combination of these loads and quantification of these loads is difficult. According to Newman (27) and Wheeler and Ackerman (28), orthodontic loads applied to tooth are 4.45N. Reynolds and von Fraunhofer stated that bond strength in the range of 5.9-7.8MPa would suffice for most orthodontic treatments (29) because maximum long-term bond strength is not intended for orthodontic treatment. Bracket bond in orthodontic treatment must be high enough to resist deboning of attachments and low enough not to damage the teeth at the time of debonding. Lopez reported that the optimal SBS for successful clinical treatment is 7MPa (30).

In the current study, SBS test was performed, which is routinely performed and has acceptable accuracy and reproducibility. The crosshead speed in the current study was 1mm/minute; crosshead speeds of 0.1-10mm/minute have been used for SBS testing; however, these values do not correspond to values in the clinical oral environment because the speed of mastication is in the range of 81-100mm/second or 4860-6000 mm/minute with a frequency of 1.03-1.2 Htz (31).

*In vitro* studies, such as the current one, have some limitations. Multifactorial oral environment cannot be accurately simulated in vitro because several factors present in the oral environment such as the saliva and patient-related behaviors and habits may affect the results. Thus, generalizability of in vitro results to the clinical setting must be done with caution. Aging is often done in vitro to better simulate the clinical setting. Thermocycling and storage in water or citric acid are also performed for further aging of composite resins *in vitro* (32,33). Moreover, some other factors such as the bonding agent used, mechanical and chemical surface preparations and type of composite can also affect the SBS of orthodontic brackets to composite surfaces. Thus, future studies are required to assess the effect of composition of composites and bonding agents on SBS values. Also, the effect of other surface preparation methods such as laser irradiation on SBS can be an interesting topic for future studies. Tensile bond strength values and bond strength of non-metallic brackets should also be evaluated.

## Conclusions

The three tested primers and the two surface preparation techniques yielded adequate SBS between orthodontic metal brackets and aged composite surfaces and were not significantly different in this respect. Considering the ARI scores, surface roughening by bur is recommended for use in the clinical setting. Also, considering the high cost of Assure Plus and Composite Primer and lack of a significant difference in SBS values of the three primers, Transbond XT seems to be a more cost-effective choice for use in the clinical setting.
